# Corneal protein repair after amniotic membrane photo-tissue bonding versus amniotic membrane graft in the treatment of corneal ulcer (an experimental study)

**DOI:** 10.1038/s41598-024-81266-5

**Published:** 2024-12-19

**Authors:** Shaimaa Mohammad, Salwa Abdelkawi, Mona Ebrahim, Aziza Ahmed, Dina Fouad

**Affiliations:** 1 Department of Medical Application of Laser, Ophthalmic Unit, National Institute of Laser Enhanced Sciences, Cairo University, Giza, Egypt; 2 Biophysics and Laser Sciences Unit, Vision Science Department, Research Institute of Ophthalmology, Giza, Egypt

**Keywords:** Corneal ulcer, Amniotic membrane photo-tissue bonding, Amniotic membrane grafting, Corneal protein, DNA fragmentation, Oxidative stress, Biophysics, Drug discovery, Medical research, Molecular medicine

## Abstract

Corneal alkali burns persist as a significant challenge in our field, often leading to a prolonged treatment course with various sight-threatening problems. This work, of utmost importance, aimed to apply the photo-tissue bonding technique (PTB) to weld the amniotic membrane (AM) to the corneal surface versus an amniotic membrane graft (AMG) and explore its safety in saving corneal protein against alkali burn.

Methods Twenty-seven rabbits with an induced corneal ulcer using 1 mol/L NaOH solution. Nine rabbits were used as an ulcer group without treatment, and the rest (n = 18) were subjected to two treatment protocols with AM. The first was attaching the AM to the corneal ulcer through photo-tissue bonding using 532 nm and rose Bengal stain as a photosynthesizing agent. The second was using cyanoacrylate glue as a tissue adhesive. The corneal total protein (TP), refractive index (RI), DNA fragmentation, and oxidative stress index (OSI) were evaluated. Results: The cornea’s TP showed a significant decrease (p˂0.001) immediately, 1 week, and 2 weeks after ulcer induction (-58.9%, -64.4%, and − 72.6%, respectively). The treatment with AM PTB showed improvement immediately (-45.2%, p˂0.001), after one week (-27.4%, p˂0.01), and after two weeks (-14.38%, p˂0.05). Moreover, the treatment with AMG showed improvement after the same periods with percentage changes of -52.05%, (p˂0.001), -41.8% (p˂0.001), and − 32.2%, (p˂0.01) with respect to the control. Moreover, RI of corneal protein showed improvement after two weeks of treatment with AM PTB (3%, p˃0.05) and AMG (7%, p˃0.05), respectively. The corneal protein DNA base pairs improved 88.49% for AM PTB and 82.35% for the AMG group. The oxidative stress was shifted towards an antioxidative state in AM PTB (-3.9%, P > 0.05) and the AMG group (15.9%, P < 0.05). Conclusion: The AM PTB technique used in corneal ulcers showed promising improvement in total corneal proteins, refractive index, DNA fragmentation, and OSI than AMG using cyanoacrylate glue. These results strongly support the use of AM PTB for ophthalmic purposes, suggesting its potential to enhance clinical research and practice for patients with corneal ulcers and ocular surface diseases.

**Supplementary Information:**

The online version contains supplementary material available at 10.1038/s41598-024-81266-5.

**Corneal protein repair after amniotic membrane photo-tissue bonding versus amniotic membrane graft in treatment of corneal ulcer (An experimental study)**.

## Background

Corneal alkali burn causes intractable keratitis, eruption formation, epithelial breakdown, inflammatory cell infiltration, stromal cell death, and endothelial dysfunction. The amniotic membrane (AM) is frequently used in cornea and sclera surgeries as a temporary patch or a reconstructive graft^1^. Amniotic membrane transplantation (AMT) covers persistent epithelial defects, pterygium surgery, and ocular surface reconstruction in stem cell deficiency. The AM comprises a single layer of epithelial cells attached to a basement membrane that lies over a stromal layer containing primarily types I and III collagen, proteoglycans, and fibroblasts. Currently, suturing the amnion to the cornea is a time-consuming process requiring high skills to place hair-fine sutures. In addition, suturing may injure the eye and act as a foreign body, leading to persistent inflammation, infection, and granuloma^1,2^.

The suture-less attachment of AM to the cornea, with its rapid formation of an immediate water-tight seal without damage to the cornea, is a reassuring sign of its efficiency^3^. Tissue adhesives, such as synthetic glue, are gaining approval in ophthalmology due to their potential to reduce the complications associated with current surgical methods. Cyanoacrylate glue, a strong tissue adhesive with high tensile strength, rapidly polymerizes upon contact with any fluid but has been associated with cytotoxicity^4^.

AM photo-tissue bonding (PTB) means sealing the AM over a sterile corneal ulcer using a light-activated technology that produces an immediate seal between tissue surfaces without additional glues or proteins^5–7^. A significant advantage of PTB is the minimal scarring and fibrosis produced compared to AM suturing^8,9^.

The Food and Drug Administration (FDA) approved Rose Bengal dye (RB) to diagnose ocular surface diseases. RB dye was used to generate singlet oxygen (^1^O_2_), which has been suggested to mediate the formation of protein − protein bonds^10,11^. Photosensitized protein crosslinking prevents deleterious changes to tissue in response to physiological pressures^12,13^. In addition, RB photosensitization makes tissue less sensitive to inflammation and is identified as photochemical tissue passivation^14^.

This work aimed to evaluate the improvement of corneal protein after treating corneal ulcers. Fortunately, all used materials, including AM, RB, and diode green laser, are FDA-approved for ophthalmic uses. This FDA approval provides a strong foundation for the safety and efficacy of the procedure. Therefore, AM PTB was applied using RB and green laser (532 nm) to weld the AM to the corneal ulcer and compared with the AMG using cyanoacrylate glue as a tissue adhesive. The corneal protein concentration, refractive index measurements, oxidative stress index, and DNA damage analysis were evaluated immediately, one week, and two weeks later.

## Results

### Total corneal protein concentration

**Fig. 1:**
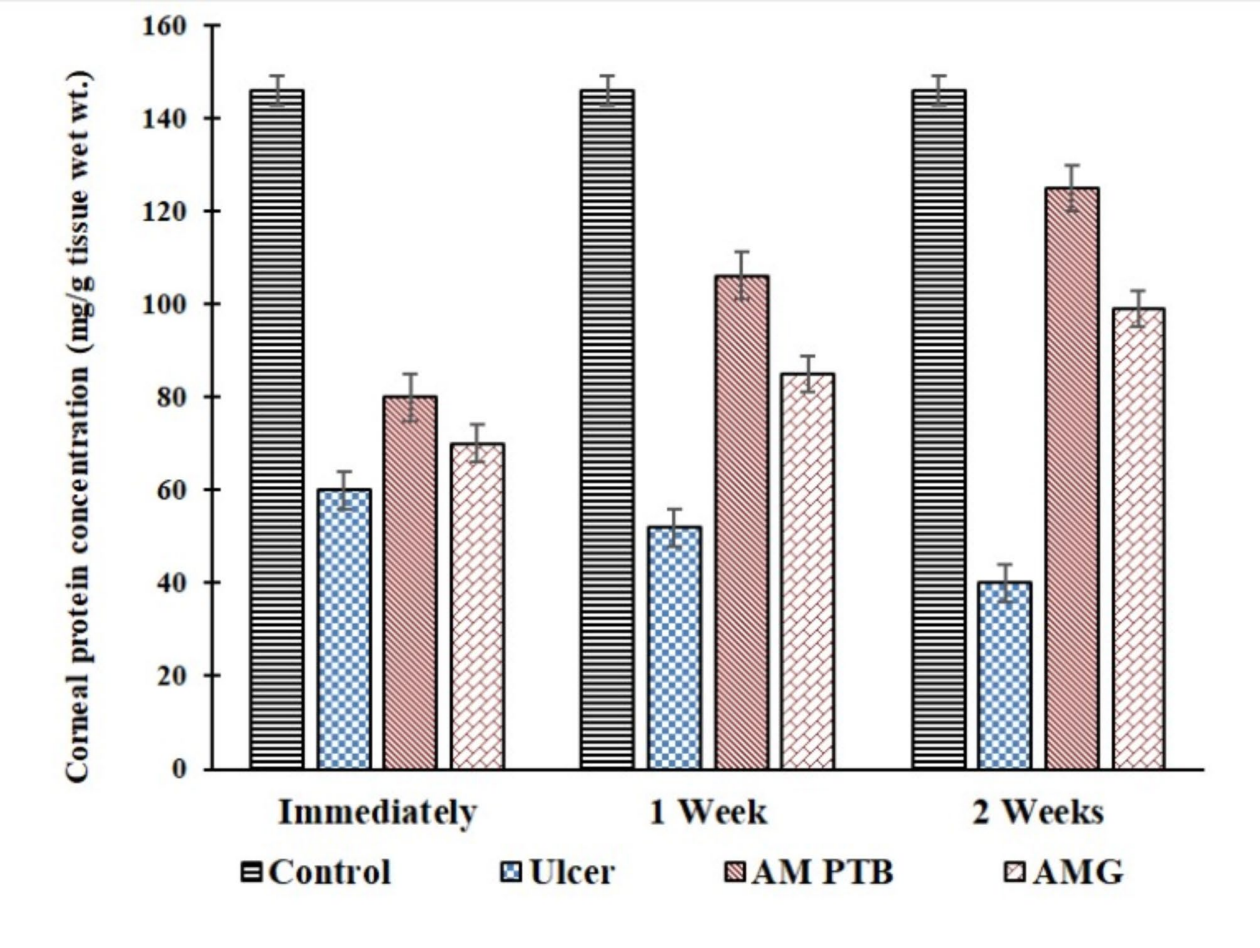
Corneal protein concentration for the control, ulcerated, and treated corneas measured immediately, 1 week, and 2 weeks. AM PTB: Amniotic membrane photo-tissue bonding, AMG: Amniotic membrane graft.

The corneal protein concentration of the ulcerated cornea and after the treatment with AM PTB and AMG is illustrated in Fig. 1. The total protein for the control was 146 ± 8 mg/g tissue wet wt and significantly decreased after induction of ulcers to 60 ± 6 mg/g tissue wet wt (p˂0.001), 52 ± 6 mg/g tissue wet wt (p˂0.001) and 40 ± 5 mg/g tissue wet wt (p˂0.001) when measured immediately, after one week and two weeks with percentage changes of −58.9%, −64.4% and − 72.6% compared with the control, respectively.Treatment of ulcers with AM PTB showed gradual improvement with values of 80 ± 6 mg/g tissue wet wt (p˂0.001), 106 ± 8 mg/g tissue wet wt (p˂0.01), and 125 ± 5 mg/g tissue wet wt (p˂0.05) with percentage changes of −45,2%, −27.4% and − 14.38% when measured immediately, after one week and two weeks respectively, compared with the control. Moreover, the treatment of the ulcer with AMG using cyanoacrylate glue showed gradual improvement after the same periods of the previous group with values of 70 ± 7 mg/g tissue wet wt (p˂0.001), 85 ± 4 mg/g tissue wet wt (p˂0.001), and 99 ± 5 mg/g tissue wet wt (p˂0.01) with percentage changes of −52.05%, −41.8% and − 32.2% with respect to the control.

## The refractive index for corneal proteins (RI)

**Fig. 2:**
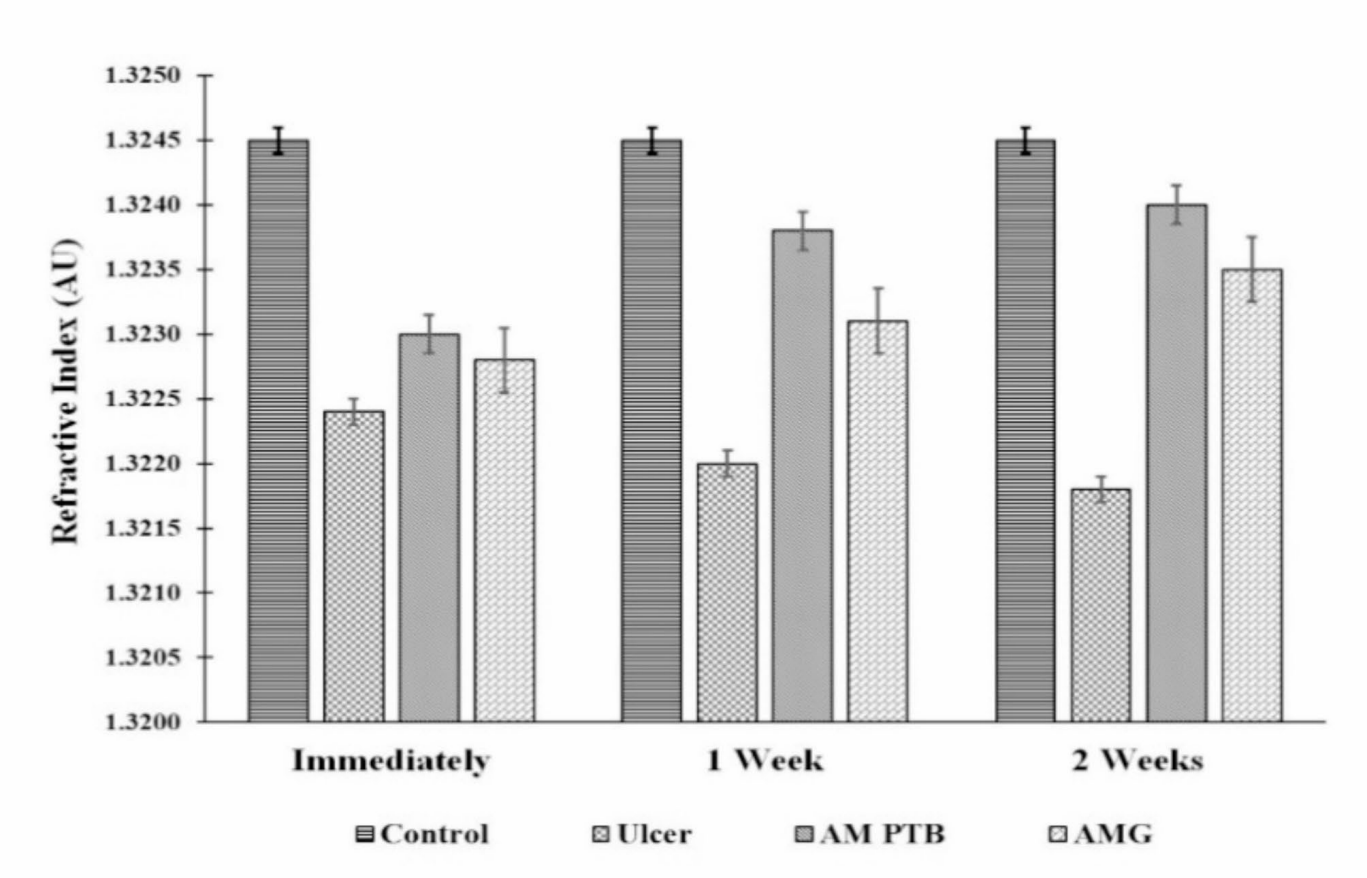
Refractive index for corneal protein immediately, 1 week and 2 weeks for the control, ulcerated, and treated corneas. AM PTB: Amniotic membrane photo-tissue bonding, AMG: Amniotic membrane graft.

The RI of corneal protein for the control, ulcerated, treated corneas with AM PTB and AMG are shown in Fig. 2. The refractive index for the sample of the control cornea was 1.3245, which showed a significant decrease immediately after induction of corneal ulcer and after one week and two weeks. The RI was 1.3224, 1.3220, and 1.3218, with percentage changes of 16%, 19%, and 20% (p˂0.01) with respect to the control. Furthermore, treatment with AM PTB showed improvement and non-significant changes in the corneal protein refractive index with values of 1.3230, 1.3238, and 1.3240 with percentage changes of 11%, 5%, and 3% (p˃0.05), respectively, when compared with the control. Moreover, the corneas treated with AMG using cyanoacrylate glue also showed improvement in the RI of corneal protein when measured immediately, one week, and two weeks with values of 1.3228, 1.3231, and 1.3235, respectively. Consequently, the percentage change compared with the control was 12%, 10%, and 7% after the three estimated periods.

## DNA fragmentation for corneal protein

**Fig. 3:**
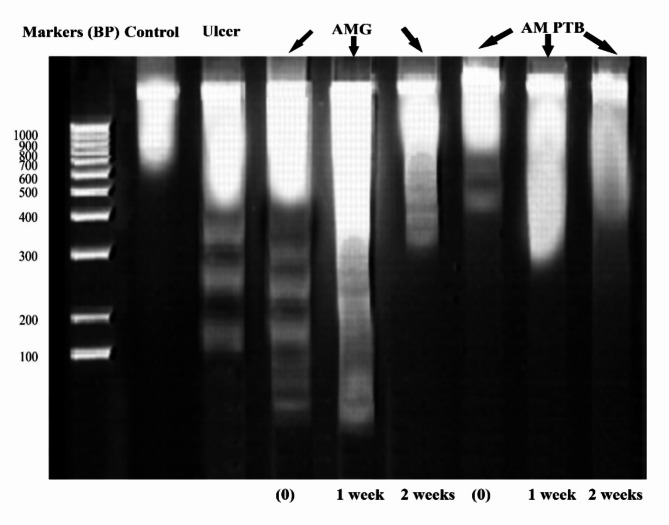
Agarose gel electrophoresis for corneal protein represents the DNA fragmentation for the control, ulcerated and treated with AM PTB and AMG after different periods. AMG: Amniotic membrane graft, PTB: Photo-tissue bonding, (0): immediately. The samples were derived from the same experiment, and the gels/blots were processed in parallel. Original blots/gels are presented in Supplementary figure S1.

The fragmentation of DNA of corneal protein was analyzed using 0.1% agarose gel electrophoresis (Fig. 3). The first lane represents the DNA markers ranging from 100 to 1000 BP (Base Pair). The second lane represents the control group in one band with 1064 BP. The third lane represents the ulcerated cornea with four fragmented bands (1151 BP, 346 BP, 256 BP, and 141 BP). The fourth lane represents the corneas treated with AMG, isolated, and immediately analyzed, which showed four fragments (1137 BP, 300 BP, 237 BP, and 152 BP). The DNA fragmentation was increased after one week (lane 5), as shown in Table 1, with values of 372 BP, 292 BP, 256 BP, 225 BP, and 200 BP. In addition, after two weeks (lane 6), there was an improvement in 82.35% of DNA but still more fragmentation in the rest of the DNA. After treatment with AM PTB (immediately), three fragments appeared in the agarose gel with 1124 BP, 636 BP, and 469 BP. The first band (1164 BP) showed noticeable improvement after two weeks, with a percentage of 88. 49% (lane 9), respectively.

**Table 1:** Corneal protein DNA fragmentation for the control, ulcer and treated with AM PTB and AMG after different periods.

Group	Lane no.	Period	Base Pair (BP)	%
**Control**	**2**		**1064**	**100**
**Corneal ulcer**	**3**		**1151** **346** **256** **141**	**60.70** **6.87** **17.36** **15.07**
**AM PTB**	**7**	**(0)**	**1124** **636** **469**	**77.91** **1.88** **20.21**
	**8**	**1 W**	**1151** **1024** **932** **874**	**72.50** **4.99** **16.38** **6.13**
	**9**	**2 W**	**1164** **824** **600**	**88.49** **6.13** **5.38**
**AMG**	**4**	**(0)**	**1137** **300** **237** **152**	**70.16** **4.73** **7.42** **17.69**
	**5**	**1 W**	**327** **292** **256** **225** **200**	**82.77** **1.38** **1.73** **3.86** **10.26**
	**6**	**2 W**	**1164** **600** **484** **406** **357**	**82.35** **3.26** **4.62** **7.09** **2.68**

**AM: Amniotic membrane**,** PTB: Photo- Tissue Bonding**,** AMG: Amniotic Membrane Grafting**,** W: Week**,** (0): Immediately**,** BP: Base Pair.**

### Evaluation of oxidative stress index (OSI).

Mean and SD values of TAC, TOC, and OSI levels for control and all treated groups were presented in Table 2. The data showed a significant elevation of TOC (114.3%, *P* < 0.001) and TAC (50.4%, *P* < 0.001) after corneal ulcer. The treatment with AM PTB showed gradual improvement in TOC and TAC after two weeks, reaching 10.7% and 11.8% with respect to the control. Alternatively, the OSI of the cornea showed non -significant changes (−3.9%, *P* > 0.05). Moreover, the treatment with AMG showed noticeable improvement in the TOC and TAC, with percentage changes of 32.1% and 13.9% values after two weeks, respectively. In the case of the corneal ulcer group, the data indicated shifting toward oxidative status (4.28 ± 0.3), and oxidative stress was very high compared with the control group (3.01 ± 0.2). After two weeks of AM PTB and AMG, the oxidative stress was shifted towards an antioxidative state with values of 2.98 ± 0.2 (−3.9%, *P* > 0.05) and 3.49 ± 0.2 (15.9%, *P* < 0.05), respectively.

**Table 2:** Oxidative stress index (OSI) of rabbit cornea for the control group, corneal ulcer group, and different groups treated with PTB and AMG.

Group	Period	TOC (mM/L)	% Change	TAC (mM/L)	% Change	OSI	% Change
Control		**0.28 ± 0.02**		**0.093 ± 0.01**		**3.01 ± 0.2**	
Corneal ulcer		**0.60 ± 0.05**	114.3^***^	**0.140 ± 0.01**	**50.4** ^***^	**4.28 ± 0.3**	**42.2** ^***^
**AM PTB**	**(0)**	**0.42 ± 0.03**	**50.0** ^***^	**0.117 ± 0.02**	**25.8** ^**^	**3.59 ± 0.2**	**19.3** ^**^
	**1 W**	**0.50 ± 0.02**	**78.6** ^***^	**0.110 ± 0.02**	**18.3** ^**^	**4.54 ± 0.3**	**50.8** ^***^
	**2 W**	**0.31 ± 0.01**	**10.7** ^*^	**0.104 ± 0.02**	**11.8** ^*^	**2.98 ± 0.2**	**− 3.9**
**AMG**	**(0)**	**0.49 ± 0.02**	**75%** ^***^	**0.106 ± 0.02**	**13.9** ^*^	**4.62 ± 0.3**	**53.5** ^***^
	**1 W**	**0.55 ± 0.02**	**96.4** ^***^	**0.116 ± 0.01**	**24.7** ^**^	**4.74 ± 0.3**	**57.5** ^***^
	**2 W**	**0.37 ± 0.01**	**32.1** ^**^	**0.106 ± 0.01**	**13.9** ^*^	**3.49 ± 0.2**	**15.9** ^*^

**TOC: Total oxidant Concentration**,** TAC: Total Antioxidant Concentration**,** PTB: Photo- Tissue Bonding**,** AMG: Amniotic Membrane Grafting**,** W: Week**, (0): Immediately. *: Statistically significant, **: High Statistically significant, ***: Very high statistically significant.

## Discussion

Our study, which utilized AM PTB on an ulcerated cornea with alkali burn, aimed to demonstrate the benefits of suture-less AM fixation to the cornea and collagen crosslinking through the photodynamic effect. Additionally, we applied the AMG technique using cyanoacrylate glue and compared the efficiency of both AM PTB and AMG in improving corneal protein. The implications of our findings are significant, as they provide valuable insights into the potential treatment of corneal ulcers, particularly in the context of alkali burns.

Previous studies used the AM PTB technique to weld or bond the edges of corneal wounds^15^. Other studies used RB and green laser-collagen crosslinking only without AM to treat a resistant infectious corneal ulcer^16,17^. Moreover, the usefulness of the fixation of AM using 2-octyl cyanoacrylate glue in treating experimental corneal burns with less vascularization and rapid epithelial healing was evaluated^18^.

Our study showed that the corneal protein concentration in the ulcerated group significantly decreased. This decrement suggests corneal melting because of the inflammatory reaction after the alkali burn. The primary mechanism of corneal melting in alkali burn is the presence of many polymorphonuclear leukocyte infiltration, collagenase, and matrix metalloproteinase, leading to the continuous digestion of corneal collagen^19^. In contrast, there was a gradual increase in the corneal proteins in the group treated with AM PTB and the group treated with AMG using cyanoacrylate glue. The improvement was significantly higher in the group treated with AM PTB than in the AMG group. The better improvement in the group of AM PTB may be due to the anti-inflammatory effect of the AM in addition to the photodynamic impact that makes the tissue less sensitive to inflammation, which is called tissue passivation^12,13^. Moreover, treatment with AM PTB reflects the increase in corneal resistance to collagenase. It prevents and delays corneal melting and can decrease collagen fibers’ damage and infiltration of inflammatory cells in the corneal tissues^20^. Furthermore, the resistance of collagen to enzymatic digestion by collagenase in corneas treated by collagen cross-linking using RB and green laser was reported previously^21^. This meets our results of the better improvement of corneal protein in the AM PTB group than the AMG group and may be due to the diffusion of RB into the stroma affecting more corneal proteins^16^. RB induces protein crosslinks without phototoxicity to keratocytes by a mechanism concerning ^1^O_2_. Further, RB forms two complexes with amnion stromal collagen, that bonding needs oxygen, and singlet oxygen mediates protein cross-linking^22^.

The corneal protein RI is crucial for characterizing proteins and complexes. This parameter is also essential, particularly for understanding the optical properties of eye tissues^23^. Empirical measurements of the corneal refractive index are limited, and the cornea’s layers exhibit varying refractive indices due to differing physiological properties^24^. Measurements of the RI of corneal protein extracts can significantly reflect the overall corneal RI from multiple structural changes rather than a single layer. In our study, the progressive decrease in RI in the ulcerated group due to reduced soluble protein in the cornea reflects improper remodeling and disordered protein structure. Furthermore, improving the RI after treatment revealed the reformation of a proper and ordered protein structure. In addition, this improvement is returned to the decrease in inflammation and melting of the cornea as reported by Soeken et al., which showed that sealing of the cornea by AM PTB could occur with minimal inflammation or secondary effects^15^.

In animal models, therapies that restore corneal transparency are evaluated using chemical and biophysical techniques. Advanced methods, such as DNA fragmentation analysis, enhance the assessment of these therapies, aiding their transition to clinical trials. Agarose gel electrophoresis outlines a method for analyzing DNA fragmentation linked to apoptosis. Key characteristics of apoptosis include DNA fragmentation, which produces a ladder pattern on the gel. While the method effectively assesses DNA fragmentation qualitatively, it allows quantitative analysis using the Gel Pro analyzer 4.5. Overall, this assay is valuable for both qualitative and quantitative evaluation of apoptosis, as shown in Table 1. Compared to the ulcerated corneas, the AMG and AM PTB-treated corneas had a less inflammatory response and effectively suppressed apoptosis (Table 1). Human amniotic epithelial cells improve the function of human corneal epithelial cells by reducing apoptosis. This is achieved through several mechanisms: decreasing reactive oxygen species (ROS) production, maintaining higher membrane potential, lowering p53 protein expression, and increasing survivin protein levels. Additionally, high expression of the PAX6 gene in corneal epithelial cells enhances cell proliferation and inhibits apoptosis, contributing to better preservation of corneal epithelial characteristics^25,26^.

Cross-linking is the formation of chemical bonds between two molecules, such as linking two DNA strands by covalent bonds. That makes the polymer chains bound together with less extent of movement^27^. They become more rigid, stronger, and more challenging as they are linked, like in our study using agarose gel electrophoresis (Fig. 3). The results revealed marked resistance of DNA to fragmentation after two weeks in both the AM PTB group (88.49%) and the AMG group (82.35%), respectively (Table 1). The improvement in the AM PTB group is revealed to the collagen crosslinking that occurs through the reaction of singlet oxygen with the surrounding amino acids, mainly histidine, to form covalent bonds across collagen fibers^20^. Oxidized histidine then reacts with specific amino acids, primarily lysine, to form protein-protein crosslinks^28^. This photochemical crosslinking can strengthen the corneal stroma, improve biomechanical rigidity, and increase corneal stiffness and resistance to collagenase digestion^12,22^. Further, the increased resistance to collagenase digestion is owed to changes in the tertiary structure of the collagen fibrils, which block the access of proteolytic enzymes to the cleavage sites through steric hindrance^29^. Steric hindrance means prevention or retardation of inter- or intramolecular interactions because of the spatial structure of a molecule. Likewise, using cyanoacrylate glue in the AMG group may prevent collagenase production with a less inflammatory response^19,30^.

The present study highlights the antioxidative and oxidative state of the corneas exposed to alkali burn and treated with AM PTB and AMG. In the corneal ulcer group, the OSI was shifted toward high oxidative status (4.28 ± 0.3) compared with the control group (3.01 ± 0.2). After two weeks of treatment, the OSI was shifted towards an antioxidative state for both groups, and the improvement was better in the AM PTB group (−3.9%, *P* > 0.05) than in the AMG group (15.9%, *P*< 0.05), with respect to the control. Reactive oxygen species (ROS) are produced via oxidative reactions during metabolic and physiological processes^31^. These ROS attack cellular components, destroying lipids, proteins, and DNA, and can initiate a chain of reactions resulting in cellular complications^32,33^. Besides, enzymatic and non-enzymatic antioxidative mechanisms have been developed to reduce ROS damage^32,33^. The endogenous and food-derived antioxidants represent the total antioxidant activity accumulated in tissues^12^. Oxidative stress results when oxidation products such as lipids and proteins increase and antioxidant enzymes and vitamins decrease^34,35^. Hence, OSI, the corneal TOC to TAC level ratio, indicates oxidative stress reflecting the redox balance between oxidation and antioxidation^11,36^. In the present study, we observed an elevation in the TOC (114.3%) and TAC (50.4%) and, alternatively, an elevation in the OSI (42.2%) after induction of corneal ulcer. The ulcer induces oxidative stress and stimulates the antioxidant defense system by elevating TAC. Furthermore, treatment with AM PTB and AMG appeared time-dependent as the results indicated gradual improvement of the cornea after the 1st and 2nd week of treatment. Excitingly, the percentage change in OSI reflects the degree of oxidative stress. Our results suggested that the oxidative/antioxidative balance shifts towards the antioxidative status in the AM PTB group (− 3.9%, *P*> 0.05), which is better than the AMG group. These results match the present results of DNA fragmentation in which the photochemical crosslinking occurred through AM PTB causes resistance to collagenase digestion, blocks the access of proteolytic enzymes to the cleavage sites through steric hindrance, prevents further damage, and gives the cell time to repair the defect^29^. This study identified the AM PTB treatment as a better choice than AMG because it improved the corneal protein, reduced DNA fragmentation, and returned the oxidative stress to a balanced state between the oxidant and antioxidant. Moreover, after the estimated periods, the healing of ulcers treated with AM PTB showed better corneal integrity than in the AMG group. The follow-up showed no significant differences between the two treatment groups. No scaring, no corneal perforation, minimal superficial punctate keratitis, and a relatively smooth corneal profile. However, there was non-significant corneal neovascularization in the AMG-treated groups after one day.

This study demonstrates that a light-activated technology using AM PTB that welds the AM to the corneal surface by forming molecular crosslinks between proteins is strong enough to seal penetrating eye wounds and requires short irradiation. The seal has the benefits of being suture-less and rapid, creating an instant water-tight seal and not triggering another injury to the cornea^17^.

The obtained data highlighted the practical implications of using a sutureless AM PTB that resulted in better corneal protein healing than AMG using cyanoacrylate glue. The results indicate that using AM PTB offers an effective method for treating corneal wounds without requiring surgical skills and reducing suture-related complications. This finding is significant as it suggests that AM PTB can be recommended as a useful option in treating corneal ulcers and ocular surface diseases, such as pterygium excision, fornix reconstruction, and corneal melting, with excellent clinical outcomes. The study’s findings on the effectiveness of AM PTB in these clinical applications provide valuable insights for medical professionals. Additionally, this paper contributes not only to comparing the two techniques but also to the importance of the measured parameters such as protein content, refractive index, DNA fragmentation, and OSI levels as valuable markers for evaluating the effectiveness of the two treatment moods.

## Methods

### Experimental animals

Thirty-six female New Zealand rabbits (weight 2.0–2.5 kg) were obtained from the animal house of the Research Institute of Ophthalmology (RIO), Giza, Egypt. The rabbits were fed a standard laboratory diet during the experiment, free access to water at 22 ± 2 °C, 50–70% humidity, and a 12 h.-day–night cycle. The study was conducted in collaboration between the National Institute of Laser Enhanced Sciences, Cairo University (NILES), and the Research Institute of Ophthalmology, Giza, Egypt.

### Induction of corneal ulcer

Adult female albino rabbits with intact corneas (*n* = 36) were divided into a control group of nine rabbits (*n* = 9), and the rest of the animals (*n* = 27) were generally anesthetized by intramuscular injection with 0.2 ml/kg xylaject^®^ (xylazine hydrochloride 2%, ADWIA, Egypt)) and ketamine hydrochloride (0.6 ml/kg). In addition, Benoxinate hydrochloride (0.4%) eye drops (Benox^®^ Eipico Co.,Cairo, Egypt.) was used for local anesthesia. A circular filter paper of 5.0 mm diameter was immersed in 1 mol/L sodium hydroxide solution (Sigma-Aldrich, St. Louis, MO, USA), centered on the animal’s right cornea for 30 s, and removed. The eye was irrigated immediately with a sterile saline solution for two minutes. The rabbits were divided equally into a corneal ulcers group (*n* = 9 eyes), a treated group with AM PTB (*n* = 9 eyes), and a treated group with AMG using cyanoacrylate glue (*n* = 9 eyes).

### Preparation of the amnion patch

The ulcerated cornea surface was flushed with RB solution (1% wt/vol in PBS, Sigma-Aldrich, St. Louis, MO, USA)) for 2 min and then briefly washed with 0.9% saline solution. The sterile human AM patch (Bio Tissue Corporation, Miami, FL 33126, USA) was placed on filter paper with the stromal surface upward. RB solution (0.1% wt/vol in PBS) was placed on the AM stromal surface for 5 min. The dye-stained amnion patch was then cut to an appropriate size to cover the corneal ulcer with the epithelial basement membrane side facing up, and wrinkles were smoothed.

### Bonding of the AM to the cornea

A diode-pumped solid-state laser was applied after the RB-stained amnion membrane was placed with its stromal surface in contact with the corneal ulcer (DPSS, HHs6-2, Tim e-relay, Elexperc, Egypt). The instrument was manufactured for research in the National Institute of Laser Enhanced Sciences (NILES), Cairo University laboratories. It emits a CW green light with a wavelength of 532 nm, irradiance of 0.25 W/cm2, and fluence of 100 J/cm^2^ through an optical fiber with a diameter of 4.0 mm.

### Amniotic membrane grafting (AMG)

A lid speculum was used to expose the cornea and prevent gluing of the lids or the nictitating membrane. The ulcer was dried and debrided with a sterile cotton swab to remove loose or devitalized epithelial tissue. Cyanoacrylate glue (Monomeric n-butyl-2-cyanoacrylate, Histoacryl^®^ B. Braun Surgical, SA, Carretera de Terrassa, Rubi, Spain) was drawn up into a 1-mL syringe through a 22-g needle. The needle was removed from the syringe, and the glue was pushed up to the flattened surface of the syringe tip and applied to cover the entire ulcerated area with a small amount extending beyond the ulcer edge. The amnion patch with an appropriate size was placed over the corneal ulcer with the epithelial basement membrane side facing up. Topical application of tobramycin/dexamethasone combination (Tobradex^®^, Alcon, USA.) was applied after finishing the procedure. Actual photographs of the various groups at different stages have been added as Supplementary figure S2.

Rabbits were euthanized with a high dose of ketamine hydrochloride and Xylazine hydrochloride (5 times the dose used in anesthesia). Three rabbits from each group were sacrificed immediately, one week, and two weeks after treatment, and the eyes were enucleated. The corneas were carefully isolated, weighed, and homogenized using a cell homogenizer (type Tübingen 7400, Germany) in a 10-fold volume of 20 mM ice-cold Tris-HCl buffer, pH 7.4. The homogenate was centrifuged for 20 min at 15,000 rpm (Awel centrifuge MS 20, France), and the supernatant was withdrawn.

### Measurement of the corneal protein concentration and the refractive index

According to Lowry et al.^37^, the supernatant was used to determine the corneal protein concentration. The corneal protein samples were measured at 750 nm using a spectrophotometer (Evolution 600 PC UV‑Vis; Thermos Fisher Scientific, Madison, WI). The corneal protein refractive index was measured using the Abbe refractometer attached to a temperature control unit at 37 ℃±0.02.

### Corneal DNA fragmentation

According to Dash et al.^38^, DNA fragmentation was evaluated using agarose gel electrophoresis. 20 µl of the DNA markers and corneal samples were loaded on 1% agarose gel wells containing 0.5 mg/ml of ethidium bromide in TBE buffer (Tris/Borate/EDTA, pH 8.0). The electrophoresis technique was run using an agarose gel electrophoresis device (Model Horizon 58, Gibco BRL, USA). The separation was viewed using a gel documentation system (Bio-Rad™) and analyzed with Gel Pro analyzer 4.5 software.

### Oxidative stress index (OSI)

The total oxidative concentration (TOC) of the cornea samples was determined using a colorimetric method^39^. The color intensity, at 510 nm, is related to the total oxidative molecules in the sample and is expressed in terms of mM/L. The total antioxidative concentration (TAC) in the supernatant of corneal samples (mM/L) was obtained using a colorimetric method at 505 nm^40^. The reaction was performed through an enzymatic reaction between the amount of antioxidants in the cornea and a defined amount of exogenously provided hydrogen peroxide (H2O2). The ratio of the total oxidative concentration (TOC) to the total antioxidative concentration (TAC) was accepted as the oxidative stress index (OSI)^41^. All reagents used were purchased from BioDiagnostic Laboratories Inc., Brooklyn, New York, USA.

### Statistical analysis

Student’s t-test for unpaired data was used to compare between groups; significance was set at *P* ≤ 0.05. The TAC, TOC, and OSI results were explained as the mean ± standard deviation.

## Electronic supplementary material

Below is the link to the electronic supplementary material.

Supplementary Material 1

Supplementary Material 2

## Data Availability

All data generated or analyzed during this study are included in this published article.

## Abbreviations

AM: Amniotic membrane
PTB: Photo tissue bonding
AMG: Amniotic membrane grafting
RB: Rose Bengal
TP: Total Protein
RI: Refractive Index
TAC: Total Antioxidative Concentration
TOC: Total Oxidative Concentration
OSI: Oxidative Stress Index
ROS: Reactive Oxygen Species
