# Supplementary material for: Corneal protein repair after amniotic membrane photo-tissue bonding versus amniotic membrane graft in the treatment of corneal ulcer (an experimental study)
Source: Sci Rep. 2024 Dec 19;14:30561. doi: 10.1038/s41598-024-81266-5 (PMC11659519; doi:10.1038/s41598-024-81266-5)
Supplement: Supplementary file 1 — Supplementary Material 1 [file 41598_2024_81266_MOESM1_ESM.pdf]

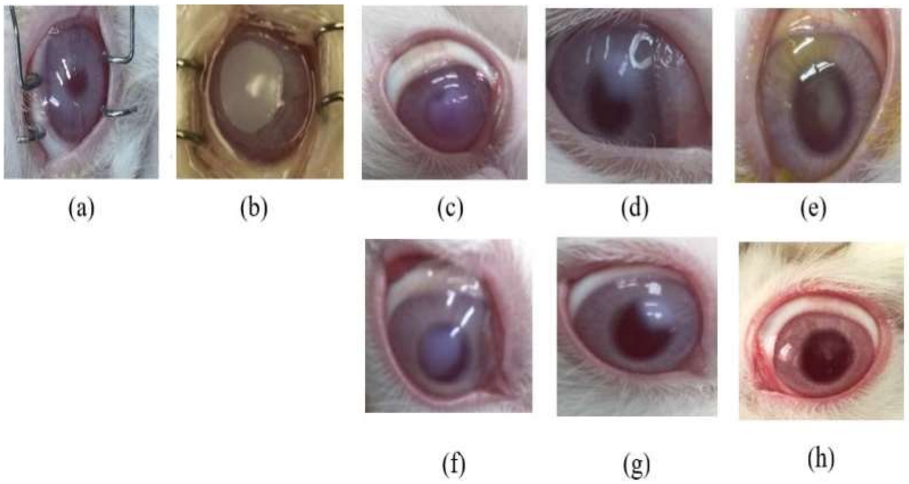

Supplementary figure S2: (a) Control eye with a wired eyelid speculum, **(b)** chemical corneal ulcer using1.0 N NaOH solution, **(c), (d), and (e):** Cornea immediately (0), one week, and two weeks of treatment with AMG, respectively; (f), (g), and (h): Cornea immediately (0), one week, and two weeks of treatment with AM PTB.
