# Supplementary material for: Corneal protein repair after amniotic membrane photo-tissue bonding versus amniotic membrane graft in the treatment of corneal ulcer (an experimental study)
Source: Sci Rep. 2024 Dec 19;14:30561. doi: 10.1038/s41598-024-81266-5 (PMC11659519; doi:10.1038/s41598-024-81266-5)
Supplement: Supplementary file 2 — Supplementary Material 2 [file 41598_2024_81266_MOESM2_ESM.pdf]

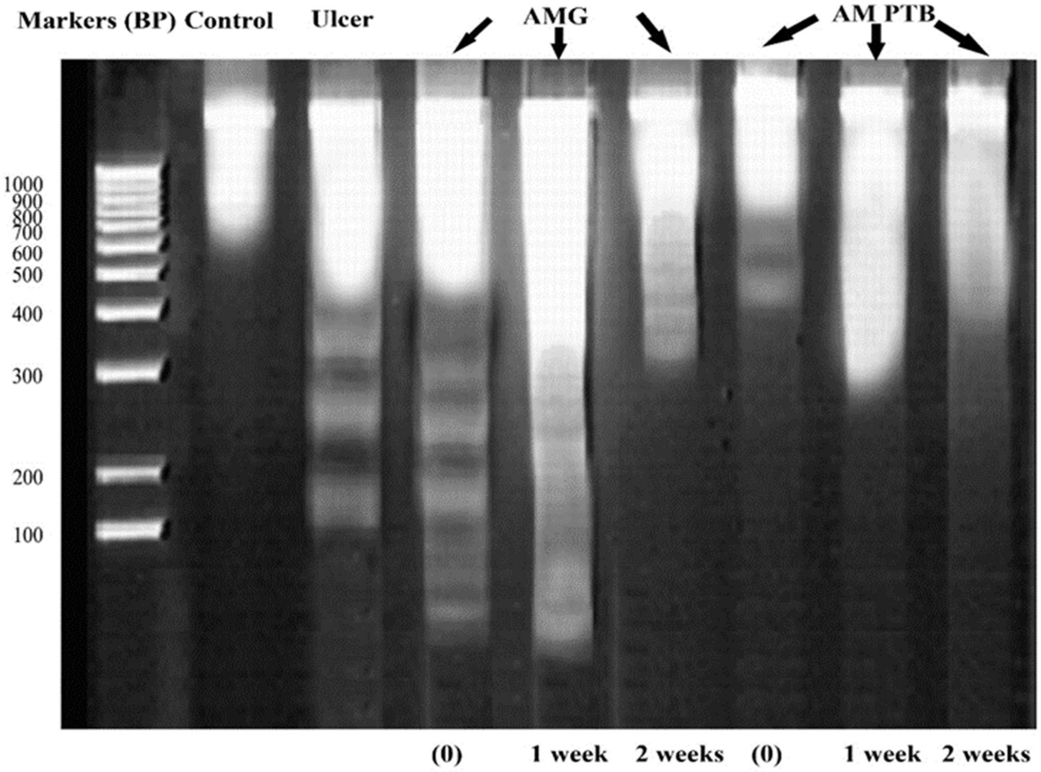

Supplementary figure S1: Agarose gel electrophoresis for corneal protein represents the DNA fragmentation for the control, ulcerated and treated with AM PTB and AMG after different periods. AMG: Amniotic membrane graft, PTB: Photo-tissue bonding, (0): immediately.
